# Supplementary figures and images for: Antimicrobial efficacy of silver nanoparticles against Candida albicans: A systematic review protocol
Source: PLoS One. 2021 Jan 25;16(1):e0245811. doi: 10.1371/journal.pone.0245811 (PMC7833133; doi:10.1371/journal.pone.0245811)

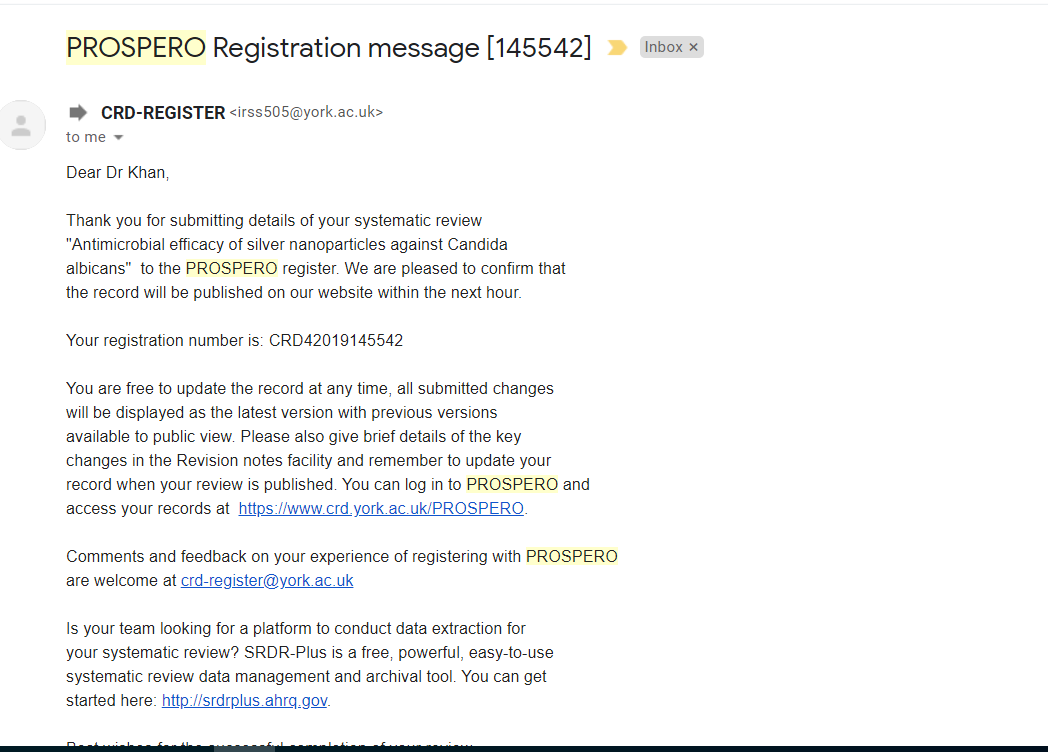

Supplement: S1 Fig — (PNG) [file pone.0245811.s002.PNG]
