# Supplementary material for: Antimicrobial efficacy of silver nanoparticles against Candida albicans: A systematic review protocol
Source: PLoS One. 2021 Jan 25;16(1):e0245811. doi: 10.1371/journal.pone.0245811 (PMC7833133; doi:10.1371/journal.pone.0245811)
Supplement: S1 File — (PDF) [file pone.0245811.s003.pdf]

Systematic review

This record cannot be edited because it is being assessed by the editorial team

1. * Review title.

Give the working title of the review, for example the one used for obtaining funding. Ideally the title should state succinctly the interventions or exposures being reviewed and the associated health or social problems. Where appropriate, the title should use the PI(E)COS structure to contain information on the Participants, Intervention (or Exposure) and Comparison groups, the Outcomes to be measured and Study designs to be included.

Antimicrobial efficacy of silver nanoparticles against Candida albicans.

2. Original language title.

For reviews in languages other than English, this field should be used to enter the title in the language of the review. This will be displayed together with the English language title.

N/A

3. * Anticipated or actual start date.

Give the date when the systematic review commenced, or is expected to commence.

02/08/2019

4. * Anticipated completion date.

Give the date by which the review is expected to be completed.

30/12/2019

5. * Stage of review at time of this submission.

Indicate the stage of progress of the review by ticking the relevant Started and Completed boxes. Additional information may be added in the free text box provided.

Please note: Reviews that have progressed beyond the point of completing data extraction at the time of initial registration are not eligible for inclusion in PROSPERO. Should evidence of incorrect status and/or completion date being supplied at the time of submission come to light, the content of the PROSPERO record will be removed leaving only the title and named contact details and a statement that inaccuracies in the stage of the review date had been identified.

This field should be updated when any amendments are made to a published record and on completion and publication of the review. If this field was pre-populated from the initial screening questions then you are not able to edit it until the record is published.

The review has not yet started: No

Review stage	Started	Completed
Preliminary searches	Yes	No
Piloting of the study selection process	No	No
Formal screening of search results against eligibility criteria	No	No
Data extraction	No	No
Risk of bias (quality) assessment	No	No
Data analysis	No	No

Provide any other relevant information about the stage of the review here (e.g. Funded proposal, protocol not yet finalised).

Protocol completed
Protocol completed

6. * Named contact.

The named contact acts as the guarantor for the accuracy of the information presented in the register record.

Saadika Khan

Email salutation (e.g. "Dr Smith" or "Joanne") for correspondence:
Dr Khan

7. * Named contact email.

Give the electronic mail address of the named contact.

skhan@uwc.ac.za

8. Named contact address

PLEASE NOTE this information will be published in the PROSPERO record so please do not enter private information

Give the full postal address for the named contact.

P.O.Box 38498\ Gatesville\ 7766

9. Named contact phone number.

Give the telephone number for the named contact, including international dialling code.

+27 721836364

10. * Organisational affiliation of the review.

Full title of the organisational affiliations for this review and website address if available. This field may be completed as 'None' if the review is not affiliated to any organisation.

University of the Western Cape

Organisation web address:
https://www.uwc.ac.za/

11. * Review team members and their organisational affiliations.

Give the title, first name, last name and the organisational affiliations of each member of the review team. Affiliation refers to groups or organisations to which review team members belong.

Dr Saadika Khan. University of the Western Cape
Dr Razia Adam. University of the Western Cape

12. * Funding sources/sponsors.

Give details of the individuals, organizations, groups or other legal entities who take responsibility for initiating, managing, sponsoring and/or financing the review. Include any unique identification numbers assigned to the review by the individuals or bodies listed.

University Research Project

13. * Conflicts of interest.

List any conditions that could lead to actual or perceived undue influence on judgements concerning the main topic investigated in the review.

None

14. Collaborators.

Give the name and affiliation of any individuals or organisations who are working on the review but who are not listed as review team members.

15. * Review question.

State the question(s) to be addressed by the review, clearly and precisely. Review questions may be specific or broad. It may be appropriate to break very broad questions down into a series of related more specific questions. Questions may be framed or refined using PI(E)COS where relevant.

In adults, does silver nanoparticles inhibit the growth of candida albicans in dentures and denture liners?

16. * Searches.

State the sources that will be searched. Give the search dates, and any restrictions (e.g. language or publication period). Do NOT enter the full search strategy (it may be provided as a link or attachment.)

PubMed, Cochrane Database, PROSPERO, EBSCOhost, Wiley and Scopus for the period 2000 to 2018.

17. URL to search strategy.

Give a link to a published pdf/word document detailing either the search strategy or an example of a search strategy for a specific database if available (including the keywords that will be used in the search strategies), or upload your search strategy.

Do NOT provide links to your search results.

dentures OR denture liners OR complete dentures OR denture acrylic OR resilient liners OR tissue conditioners AND silver nanoparticles AND candida albicans AND period 2000 to 2018

Do not make this file publicly available until the review is complete

18. * Condition or domain being studied.

Give a short description of the disease, condition or healthcare domain being studied. This could include health and wellbeing outcomes.

Oral candidiasis is a common oral condition affecting more than 70% of denture wearers.It is routinely treated with oral hygiene instruction, placement of tissue conditioners and systemic antifungals. The over prescribing of antimicrobials and the development of resistant infections demands a need for alternative treatment regimens.Silver nanoparticles are a new and novel method of treating infections.

19. * Participants/population.

Give summary criteria for the participants or populations being studied by the review. The preferred format includes details of both inclusion and exclusion criteria.

Studies that report on male and/ or female human participants as well as animal studies, in vitro

20. * Intervention(s), exposure(s).

Give full and clear descriptions or definitions of the nature of the interventions or the exposures to be reviewed.

Any synthesised silver nanoparticles

21. * Comparator(s)/control.

Where relevant, give details of the alternatives against which the main subject/topic of the review will be compared (e.g. another intervention or a non-exposed control group). The preferred format includes details of both inclusion and exclusion criteria.

Complete dentures with no modifications

22. * Types of study to be included.

Give details of the types of study (study designs) eligible for inclusion in the review. If there are no restrictions on the types of study design eligible for inclusion, or certain study types are excluded, this should be stated. The preferred format

includes details of both inclusion and exclusion criteria.

In vitro studies, human and animal studies will be included where these have investigated silver nanoparticles in prosthodontics.

23. Context.

Give summary details of the setting and other relevant characteristics which help define the inclusion or exclusion criteria.

Context and setting includes clinical dental treatment and/ or any laboratory studies where this material was researched.

24. * Main outcome(s).

Give the pre-specified main (most important) outcomes of the review, including details of how the outcome is defined and measured and when these measurement are made, if these are part of the review inclusion criteria.

Primary outcomes:
Resolution of denture stomatitis

Timing and effect measures
1 year

25. * Additional outcome(s).

List the pre-specified additional outcomes of the review, with a similar level of detail to that required for main outcomes. Where there are no additional outcomes please state 'None' or 'Not applicable' as appropriate to the review

Secondary outcomes:
Efficacy of silver nanoparticles on fungal activity
Effect of silver nanoparticles on denture stomatitis
Effect of silver nanoparticles on candida albicans

Timing and effect measures
1 year

26. * Data extraction (selection and coding).

Describe how studies will be selected for inclusion. State what data will be extracted or obtained. State how this will be done and recorded.

Clinical dental treatment and/ or any laboratory studies where this material was researched. Study characteristics, where research conducted, types of materials used, outcomes related to materials, any tools or instrumentation used are data that will be extracted. Specialized tables and data synthesis, where appropriate (meta-analysis) will be completed

27. * Risk of bias (quality) assessment.

Describe the method of assessing risk of bias or quality assessment. State which characteristics of the studies will be assessed and any formal risk of bias tools that will be used.

For specific study designs, the appropriate tools will be used. For example, for randomized controlled studies, the Cochrane Risk of Bias tool will be used.

28. * Strategy for data synthesis.

Provide details of the planned synthesis including a rationale for the methods selected. This **must not be generic text** but should be **specific to your review** and describe how the proposed analysis will be applied to your data.

We would like to do a meta analysis for studies with similar comparisons and reporting the same outcomes. We will combine mean differences for continuous outcomes, and relative risks using fixed-effect or random-effects models when appropriate.

29. * Analysis of subgroups or subsets.

State any planned investigation of 'subgroups'. Be clear and specific about which type of study or participant will be included in each group or covariate investigated. State the planned analytic approach.

If there are major differences between studies accessed, we may place them in different groups specific for this research. For example, we may group laboratory studies for silver nanoparticles, silver nanoparticle treatment of denture stomatitis; silver nanoparticle and fungal activity to examine heterogeneity.

30. * Type and method of review.

Select the type of review and the review method from the lists below. Select the health area(s) of interest for your review.

Type of review

Cost effectiveness	No
Diagnostic	No
Epidemiologic	No
Individual patient data (IPD) meta-analysis	No
Intervention	Yes
Meta-analysis	Yes
Methodology	No
Narrative synthesis	Yes
Network meta-analysis	No
Pre-clinical	No
Prevention	No
Prognostic	No
Prospective meta-analysis (PMA)	No
Review of reviews	No
Service delivery	No
Synthesis of qualitative studies	No
Systematic review	Yes
Other	No

Health area of the review

Alcohol/substance misuse/abuse	No
Blood and immune system	No
Cancer	No
Cardiovascular	No
Care of the elderly	Yes
Child health	No
Complementary therapies	Yes
Crime and justice	No

Dental	Yes
Digestive system	No
Ear, nose and throat	No
Education	No
Endocrine and metabolic disorders	No
Eye disorders	No
General interest	No
Genetics	No
Health inequalities/health equity	No
Infections and infestations	Yes
International development	No
Mental health and behavioural conditions	No
Musculoskeletal	No
Neurological	No
Nursing	No
Obstetrics and gynaecology	No
Oral health	Yes
Palliative care	No
Perioperative care	No
Physiotherapy	No
Pregnancy and childbirth	No
Public health (including social determinants of health)	No
Rehabilitation	No
Respiratory disorders	No
Service delivery	No
Skin disorders	No
Social care	No
Surgery	No
Tropical Medicine	No

Urological	No
Wounds, injuries and accidents	No
Violence and abuse	No

31. Language.

Select each language individually to add it to the list below, use the bin icon to remove any added in error.

English

There is not an English language summary

32. Country.

Select the country in which the review is being carried out from the drop down list. For multi-national collaborations select all the countries involved.

South Africa

33. Other registration details.

Give the name of any organisation where the systematic review title or protocol is registered (such as with The Campbell Collaboration, or The Joanna Briggs Institute) together with any unique identification number assigned. (N.B. Registration details for Cochrane protocols will be automatically entered). If extracted data will be stored and made available through a repository such as the Systematic Review Data Repository (SRDR), details and a link should be included here. If none, leave blank.

N/A

34. Reference and/or URL for published protocol.

Give the citation and link for the published protocol, if there is one

N/A

No I do not make this file publicly available until the review is complete

35. Dissemination plans.

Give brief details of plans for communicating essential messages from the review to the appropriate audiences.

Presentation at a Conference and then subsequent Publication of the Review

Do you intend to publish the review on completion?

Yes

36. Keywords.

Give words or phrases that best describe the review. Separate keywords with a semicolon or new line. Keywords will help users find the review in the Register (the words do not appear in the public record but are included in searches). Be as specific and precise as possible. Avoid acronyms and abbreviations unless these are in wide use.

Silver nanoparticles, fungal infections, candida, denture liners, Risk of Bias assessment

37. Details of any existing review of the same topic by the same authors.

Give details of earlier versions of the systematic review if an update of an existing review is being registered, including full bibliographic reference if possible.

N/A

38. * Current review status.

Review status should be updated when the review is completed and when it is published. For newregistrations the review must be Ongoing.

Review_Ongoing

39. Any additional information.

Provide any other information the review team feel is relevant to the registration of the review.

Nanotechnology application in dental research is fairly novel in South Africa

40. Details of final report/publication(s).

This field should be left empty until details of the completed review are available.
