# Supplementary material for: Antimicrobial efficacy of silver nanoparticles against Candida albicans: A systematic review protocol
Source: PLoS One. 2021 Jan 25;16(1):e0245811. doi: 10.1371/journal.pone.0245811 (PMC7833133; doi:10.1371/journal.pone.0245811)
Supplement: Attachment — Submitted filename: REBUTTAL. Journal Comments. Nov 2020.docx [file pone.0245811.s004.docx]

Journal Requirements:

When submitting your revision, we need you to address these additional requirements.

1. Please ensure that your manuscript meets PLOS ONE's style requirements, including those for file naming. The PLOS ONE style templates can be found at

<https://journals.plos.org/plosone/s/file?id=wjVg/PLOSOne_formatting_sample_main_body.pdf> and

<https://journals.plos.org/plosone/s/file?id=ba62/PLOSOne_formatting_sample_title_authors_affiliations.pdf>

2. Please amend either the title on the online submission form (via Edit Submission) or the title in the manuscript so that they are identical.

3. Please include a separate caption for each figure in your manuscript. Thank you. Completed.

4. Please include your tables as part of your main manuscript and remove the individual files. Please note that supplementary tables should be uploaded as separate "supporting information" files.

Thank you. This has been completed.

5. Please include captions for your Supporting Information files at the end of your manuscript, and update any in-text citations to match accordingly. Please see our Supporting Information guidelines for more information: http://journals.plos.org/plosone/s/supporting-information.

Thank you. This has been corrected and completed.

1. Does the manuscript provide a valid rationale for the proposed study, with clearly identified and justified research questions?
The research question outlined is expected to address a valid academic problem or topic and contribute to the base of knowledge in the field.

Reviewer #1: Partly …. The rationale has been explained in detail under the section “Why is it important to do this review” on page 5 of the Protocol manuscript.

The research question has been expressed in detail using the PICO format on Page 6 of this Protocol

Reviewer #2: Yes

Reviewer #3: Yes

2. Is the protocol technically sound and planned in a manner that will lead to a meaningful outcome and allow testing the stated hypotheses?

The manuscript should describe the methods in sufficient detail to prevent undisclosed flexibility in the experimental procedure or analysis pipeline, including sufficient outcome-neutral conditions (e.g. necessary controls, absence of floor or ceiling effects) to test the proposed hypotheses and a statistical power analysis where applicable. As there may be aspects of the methodology and analysis which can only be refined once the work is undertaken, authors should outline potential assumptions and explicitly describe what aspects of the proposed analyses, if any, are exploratory.

 Reviewer #1: Yes

Reviewer #2: Partly The format used for this systematic review follow the detailed guidelines provided by the Cochrane Collaboration for conducting systematic reviews (REF: Higgins JPT, Green S. Cochrane Handbook for systematic reviews of interventions. 1^st^ Edition. West Sussex, UK: Wiley-Blackwell 2009).

Reviewer #3: Yes

3. Is the methodology feasible and described in sufficient detail to allow the work to be replicable?

 Reviewer #1: Yes

Reviewer #2: No The format used for this systematic review follow the detailed guidelines provided by the Cochrane Collaboration for conducting systematic reviews (REF: Higgins JPT, Green S. Cochrane Handbook for systematic reviews of interventions. 1^st^ Edition. West Sussex, UK: Wiley-Blackwell 2009).

Reviewer #3: Yes

4. Have the authors described where all data underlying the findings will be made available when the study is complete? Yes, a data availability statement is included at the end of the manuscript.

The [PLOS Data policy](https://journals.plos.org/plosone/s/materials-and-software-sharing) requires authors to make all data underlying the findings described in their manuscript fully available without restriction, with rare exception, at the time of publication. The data should be provided as part of the manuscript or its supporting information, or deposited to a public repository. For example, in addition to summary statistics, the data points behind means, medians and variance measures should be available. If there are restrictions on publicly sharing data—e.g. participant privacy or use of data from a third party—those must be specified.

 Reviewer #1: Yes

Reviewer #2: No Yes, a data availability statement has now been included at the end of the

Reviewer #3: No	manuscript.

**Reviewer #1**: Review comments
Background
1st paragraph, 5th line, in 2013, it was reported that … comment where was it reported (in Africa, the US etc or where) … in low and middle income countries globally (REF: Tyrovolas S, Koyanagi A, Panagiotakos DB, Haro JM, Kassebaum NJ, Chrepa V, et al. Population prevalence of edentulism and its association with depression and self-rated health. Sci Rep. 2016; 6: 37083.
Line 9… lack of access or limited access to dental care is strongly associated with edentulism (provide a source to factual statements). (REF: Tyrovolas S, Koyanagi A, Panagiotakos DB, Haro JM, Kassebaum NJ, Chrepa V, et al. Population prevalence of edentulism and its association with depression and self-rated health. Sci Rep. 2016; 6: 37083. The reference has been added

2nd paragraph line 4 the imbalance of oral microbes in the oral cavity is then…source?
REF: Lewis MAO, Williams DW. Diagnosis and management of oral candidosis. Br Dent J 2017; 223: 675–681. doi:10.1038/sj.bdj.2017.886. The reference has been added
3rd paragraph line 2nd… Candida Albicans is the most common causative pathogen of oral…source?

The reference has been added

7th paragraph (Why is it important to do this systematic review?), 1st line…There is no standard of care in the management of denture stomatitis…source?

REF: Lima, J.F.M., Maciel, J.G., Arrais, C.A.G.C.A.G., Porto, V.C.V.C., et al. (2016) Effect of incorporating antifungals on the water sorption and solubility of interim resilient liners for denture base relining. Journal of Prosthetic Dentistry. [Online] 115 (5), 611–616.

8th paragraph, 5th line: Nanomedicine refers to the use of nanotechnology in the diagnosis of disease...source? The references have been added

The gap statement on page 5, 3rd paragraph, line 7 to 9 does not match with the aim of the study. 1. will this systematic review harmonize studies by looking at the effect of nanotechnologies on the human body or it will only look at silver nanoparticles on the human body? 2. kindly draft the gap statement such that, it is clear to understand the knowledge gap and what to expect in this study.
If the systematic review aims to determine if silver nanoparticles inhibit the growth of candida albicans, then there should be a literature/ knowledge gap established and it is for this reason you embark on the systematic review to extend knowledge in this area.

The SR is investigating the effect of silver nanoparticles in the oral environment: this statement was rephrased. Thank you for such detailed reviewing. I hope this explains the gap in knowledge being investigated.
Objective
Have a SMART objective free from abbreviations. Kindly write SR and AgNPs in full

Thank you. This has been corrected.

Methods
Page 9, 3rd paragraph, 5th line …first write in full the name of the University (UWC) before you can later abbreviate. Thank you. The University name was written in full on Page 6, Methods. But I omitted the abbreviation (UWC) at that stage. This has now been added on page 6.

Grammar
Efficiency should be the efficiency, introduce an article “the” Thank you. This has been corrected.
Early loss should be the early loss Thank you. This has been corrected.
“is” should be changed to “are” Denture hygiene, appropriate mouth rinses and the use of …. “are” instead of “is” Thank you. This has been corrected.
Nontoxic should be hyphenated .. Thank you. This has been corrected.
The use of these chemicals may limit… you are referring to several chemicals hence the statement should be changed to “their” instead of “its” Thank you. This has been corrected.
The biological methods are cost-effective, environmental friendly and includes….change “includes” to “include”. Thank you. This has been corrected.
Page 4, 2nd paragraph, line 6; the synthesis of silver nanoparticles…replace “have been shown to be to “are” Thank you. This has been corrected.
Page 5, 1st paragraph 1st line introduce an article “the” before the word infected denture. Thank you. This has been corrected.
page 11, point number 6 (other bias) line 3…each of the 6 criteria are…replace “are” with “is”…NB: kindly, pay attention to subject-verb agreement .. Thank you. This has been corrected.
point number 6 (other bias) line 6…criteria was…replace “was” with “were” Thank you. This has been corrected.
page 12 last paragraph, 1st line, “as guide” should be changed to “as a guide” Thank you. This has been corrected.

**Reviewer #2:**

The manuscript aims at determining if Silver nanoparticles inhibit the growth of Candida Albicans when included in acrylic dentures and in different denture liners. However, the following are my comments:
Major Comments:
The abstract needs to be rewritten to be able to stand alone. It should include brief introduction, methods and analysis, and expected results. Thank you. This has been corrected.

In the background authors identified that imbalance of oral microbes in the oral cavity is affected by advanced age. Hence, the age of the participants must be clearly stated. Also, why is the inclusion of animal studies here? Thank you. This has been corrected. I have now excluded animal studies.

The PRISMA-P must be strictly followed to drive home the points the protocol set to deliver. Thank you. I agree wholeheartedly.

For search strategy, I think the draft must include at least one well defined electronic database e.g. PubMed that will be replicated for other databases. Thank you. This has been added on page 9; and will be used in PubMed when we set out to search.

No concluding remarks. The authors should include conclusion to summarise the findings of the protocol. Thank you. Yes, I agree, and this has now been added.

Minor Comments
The manuscript needs to be properly proofread … Thank you. This has been completed.
The background should be more detailed … Thank you.

We have tried to keep the focus on the subject matter being reviewed and included a literature on the area where research is conducted as it impacts on this context.

**Reviewer #3:** The authors wrote an excellent protocol for the systematic review on the use AgNPs technology in denture and denture liners and its efficacy against Candida albicans. Thank you for your kind words. Your input is truly appreciated.

A few suggestions for improvement and some questions I have are listed below:

1. While the author made their case in a sensible manner as to the significance of conducting this systematic review, It is essential that the claims made on the back ground and significance section be referenced with appropriate literature. Thank you. This has been corrected now.
E.g.1“The lack of access or limited access to dental care is strongly associated with edentulism. In low-income countries, early loss of teeth due to extractions are common. Extraction of the offending tooth is often the only acceptable, affordable and available option to treat carious teeth for these communities”. Thank you. This has been corrected and referenced.
E.g.2 “Topical antifungal therapy such as the use of Nystatin is commonly used in the management of denture stomatitis. However, recurrent infections are common as well as the potential for the development of antifungal resistance. The use of topical antifungals is further complicated by the continuous removal of antifungal action by saliva and swallowing, lack of patient compliance and the persistent contact between infected denture base and affected tissues.”

While introducing the research question with a PICO method, the authors used the term Adult in the beginning of the question. Is this in reference to human trials? If so it will be helpful for the reader if it is also reflected in the sample/participants section. Thank you. I agree. This has been corrected.

2. On electronic search, The author expressed that search strategies will be tailored according specific data bases, is author referring that this will also insure de-duplication or has the author plans to undergo a deduplication process after search? I suggest the author clarify this in the text.

Thank you. Yes, that is correct and we have changed the wording accordingly.

3. While the authors presented a compelling discussion on the significance of conducting the systematic review and what it can contribute on this particular area of science in the discussion section, There was no discussion of the potential challenges/limitations. It will be help full for the readers to discuss if there are any potential limitations to conduct the systematic review.

How does the authors plan to pull findings from human studies and laboratory studies? Does the variation in the study types pause a challenge while pulling the findings? Thank you. Yes, I agree. These areas have been rephrased, added and addressed.
